# A History of Burn Care

**DOI:** 10.3390/medicina57060541

**Published:** 2021-05-28

**Authors:** Lars-Peter Kamolz, Bernd Hartmann

**Affiliations:** 1 Division of Plastic, Aesthetic and Reconstructive Surgery, Department of Surgery, Medical University Graz, 8036 Graz, Austria; 2 COREMED—Cooperative Center for Regenerative Medicine, Joanneum Research Forschungsgesellschaft mbH, 8010 Graz, Austria; 3 Burn Center and Plastic Surgery, Unfallkrankenhaus Berlin, 12683 Berlin, Germany; bernd.hartmann@ukb.de

Burn injuries are still one of the most common and devastating injuries in humans and the treatment of major burns remains a major challenge for physicians worldwide. Modern burn care involves many components from initial first aid, burn size and burn depth assessment, fluid resuscitation, wound care, excision and grafting/coverage, infection control and nutritional support. 

Progress in each of these areas has contributed significantly to the overall enhanced survival of burn victims over the past decades. 

In this Special Issue we look back at how the treatment of burns has evolved over the past decades and hundreds of years. Most major advances in burn care occurred in the last 50 years, spurred on by wars and great fires. The use of systemic antibiotics and topical anti-infective agents greatly reduced sepsis-related mortality. This, along with the improvement of new surgical and skin-grafting techniques, allowed the earlier excision and coverage of deep burns which resulted in greatly improved survival rates and better functional and aesthetic outcomes. Advancements concerning objective burn assessment paved the way for a more accurate fluid resuscitation, minimising the effects of shock and avoiding fluid over-resuscitation. 

This article aims to explore the history of burn care to identify milestones and step-changes in each of these areas in the patient’s care and burn care-related research. The advancement of burn care has been closely associated with our deeper understanding of its pathophysiology; we have now come to understand the impact that burn injuries have in multiple fields of current medical science i.e., in metabolism and circulation, electrolyte balance and nutrition, immunology and infection, inflammation, pulmonary function and wound healing. Despite this, many challenges still remain and the focus of burn care in the future will be to overcome the existing problems of burn-related injuries (e.g., burns in the elderly, extensive burn injuries, shortening healing times and, therefore, lengths of hospital stay, and to improve scarring). It is hoped that new technologies and advances in wound care will achieve faster wound coverage with minimal scarring.

We invite you to read the articles of “A History of Burn Care” in order to learn from the past and to be fit for the future in burn care.

